# Specific Dysregulation of IFNγ Production by Natural Killer Cells Confers Susceptibility to Viral Infection

**DOI:** 10.1371/journal.ppat.1004511

**Published:** 2014-12-04

**Authors:** Nassima Fodil, David Langlais, Peter Moussa, Gregory Allan Boivin, Tania Di Pietrantonio, Irena Radovanovic, Anne Dumaine, Mathieu Blanchette, Erwin Schurr, Philippe Gros, Silvia Marina Vidal

**Affiliations:** 1 Department of Human Genetics and Department of Microbiology and Immunology, McGill University, Life Sciences Complex, Montreal, Quebec, Canada; 2 Biochemistry Department, McGill University, Montréal, Québec, Canada; 3 Research Institute of the McGill University Health Centre, McGill Centre for the Study of Host Resistance, Department of Medicine, McGill University, Montreal, Quebec, Canada; 4 McGill Centre for Bioinformatics and School of Computer Science, McGill University, Montréal, Québec, Canada; Oregon Health & Science University, United States of America

## Abstract

Natural Killer (NK) cells contribute to the control of viral infection by directly killing target cells and mediating cytokine release. In C57BL/6 mice, the Ly49H activating NK cell receptor plays a key role in early resistance to mouse cytomegalovirus (MCMV) infection through specific recognition of the MCMV-encoded MHC class I-like molecule m157 expressed on infected cells. Here we show that transgenic expression of Ly49H failed to provide protection against MCMV infection in the naturally susceptible A/J mouse strain. Characterization of Ly49H^+^ NK cells from *Ly49h*-A transgenic animals showed that they were able to mount a robust cytotoxic response and proliferate to high numbers during the course of infection. However, compared to NK cells from C57BL/6 mice, we observed an intrinsic defect in their ability to produce IFNγ when challenged by either m157-expressing target cells, exogenous cytokines or chemical stimulants. This effect was limited to NK cells as T cells from C57BL/6 and *Ly49h*-A mice produced comparable cytokine levels. Using a panel of recombinant congenic strains derived from A/J and C57BL/6 progenitors, we mapped the genetic basis of defective IFNγ production to a single 6.6 Mb genetic interval overlapping the *Ifng* gene on chromosome 10. Inspection of the genetic interval failed to reveal molecular differences between A/J and several mouse strains showing normal IFNγ production. The chromosome 10 locus is independent of MAPK signalling or decreased mRNA stability and linked to MCMV susceptibility. This study highlights the existence of a previously uncovered NK cell-specific *cis*-regulatory mechanism of *Ifnγ* transcript expression potentially relevant to NK cell function in health and disease.

## Author Summary

Cytomegalovirus (CMV) is a ubiquitous herpesvirus that largely infects the human population leading to a significant cause of disease and death in the immunocompromised and elderly. The study of CMV in animal models has helped understand the pathogenic consequences of CMV infection and adds substantial understanding of the complex interplay of host and virus in living systems. Natural Killer (NK) cells have emerged as an important player during CMV infection trough their specific recognition of viral particles determinants and subsequent secretion of cytokines and cytolytic granules. In the present study, we have generated different mouse models to specifically investigate quantify viral recognition and cytokine expression by NK cells during CMV infection as a measure of NK cell function. We found that even after proper recognition of infected cells by NK cells, the adequate production of IFNγ is crucial to restrain viral infection. Moreover, we demonstrated that IFNγ production by NK cells is genetically determined and directly linked to the IFNγ locus. Hence, we provide the first evidence for of a unique mechanism of IFNγ production by NK cells which regulates susceptibility to viral infection.

## Introduction

Natural killer (NK) cells are pivotal for both the destruction of virally-infected cells and for the cytolysis of certain tumor cells [1]. These processes are dependent on the interaction of NK cell receptors with their cognate ligands on target cells. NK cell responses are controlled by the integration of multiple triggering signals from families of cell-surface-activating and -inhibitory NK receptor such as mouse Ly49 molecules and human p58, or killer cell immunoglobulin-like receptors (KIRs) [2], [3]. Activating NK cell receptors detect specific pathogen–associated structures. These receptors lack an intracellular signaling domain and associate non-covalently with the immunoreceptor tyrosine-based activation motif–containing adaptor DAP12, CD3ξ or FcεRIγ or the Tyr-Ile-Asn-Met motif–containing adaptor DAP10 [4]. Engagement of activating receptors results in cytoskeletal rearrangement, proliferation and the secretion of lytic granules and cytokines. Conversely, inhibitory receptors possess tyrosine-based inhibitory motifs (ITIM) in their intracellular domains. MHC class I ligation induces ITIM phosphorylation and the subsequent recruitment of the tyrosine phosphatases SHP-1 and SHP-2. These dephosphorylate downstream signaling molecules that are required for activating responses.

Extensive evidence has demonstrated that host MHC-I expression heavily affects NK cell responsiveness; defects in NK cell-dependent target cell killing, rejection of allogeneic bone marrow, and IFNγ production are observed in MHC-I deficient mice [5]. This hyporesponsiveness has been attributed to dampened stimulatory signaling [6], [7]. Indeed, NK cell function which include killing and cytokine production were restored upon re-introduction of an MHC-I molecule, however, only on NK cells that carry a cognate inhibitory receptor for the MHC-I molecule. These competent NK cells were therefore called “armed” or “licensed” [8], [9].

Genetic analyses evaluating strain-dependent differences in the response to MCMV infection have provided detailed insight into NK cell activation and recognition of infected cells. In C57BL/6 (B6) mice, NK cells express the Ly49H receptor, which recognizes the viral m157 glycoprotein, a MHC class I molecule expressed on the surface of infected cells [10]. Mice lacking a *Ly49h* gene or harbouring a non-functional DAP12 adaptor molecule are susceptible to MCMV infection [11], [12]. Conversely, transgenic expression of *Ly49h* in an otherwise genetically susceptible strain imparts resistance to MCMV [13]. Engagement of the m157/Ly49h complexes triggers a number of downstream signalling events and effector functions. Firstly, NK cells mediate contact dependent cytotoxicity through the release of perforin (Prf) and granzymes (Gzms) [14]. Prf facilitates the entry and trafficking of Gzm proteases into target cells, which ultimately leads to DNA fragmentation and cell death. NK cells also secrete cytokines, such as IFNγ, during the acute phase of MCMV infection. It has been well documented that IFNγ inhibits both MCMV and HCMV viral replication *in vitro* [15], [16]. Moreover, deficiency in IFNγ has been associated with high mortality and lack of viral clearance upon infection with MCMV, emphasizing its important role in protection against MCMV induced lethality and pathogenesis [17].

In the present study, we investigated the effect of the host genetic background on NK cell responses and addressed whether acquisition of Ly49H magnifies NK cell antiviral activity. Furthermore, we carried out a genetic screen to identify mechanisms that regulate IFNγ production by NK cells. These studies demonstrate that IFNγ production in the context of Ly49H is critical for NK cell anti-viral function. Moreover, they uncover a previously unknown regulatory mechanism of *Ifng* transcription in NK cells that correlates with increase susceptibility to CMV infection.

## Results

### Ly49H expression by NK cells does not rescue mice from death upon MCMV infection

**Figure 1:**
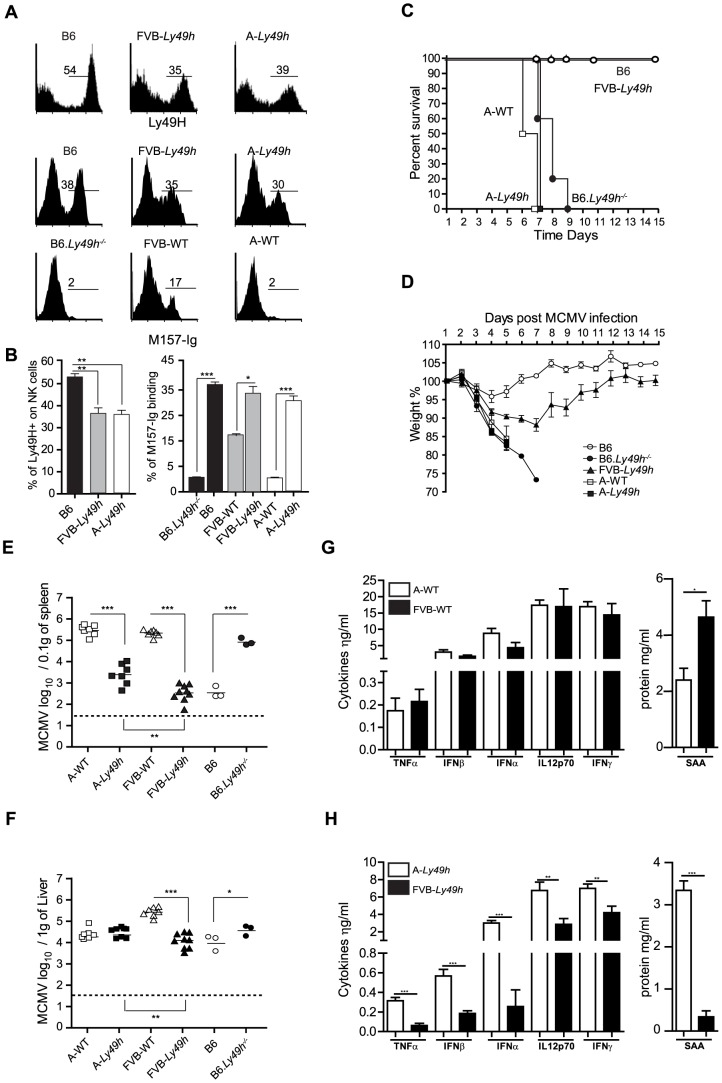
Ly49H expression by NK cells does not rescue A/J mice from lethal MCMV infection. (A) Expression of Ly49H (top) and binding of M157-Ig (bottom) gaited on CD3^−^DX5^+^ NK cells. (B) Quantification of Ly49H staining or M157-Ig binding in indicated strains. Data are representative of one experiment out of 2 (B6: n = 3, FVB-*Ly49h*: n = 3, A-*Ly49h* n = 3). (C, D) Survival and weight loss following infection with 7000 PFU (high dose) of MCMV. Two independent experiments pooled together are shown (B6, n = 10; FVB-*Ly49h*, n = 8; A-WT, n = 6, A-*Ly49h,* n = 10, B6-*Ly49h^−/−^,* n = 8). (E, F) Viral titer in the spleen and liver of mice infected with 2500PFU (Low dose) of MCMV for 3 days. Data are presented as mean ± SEM and significant *P* values are indicated. (G, H) Serum from Wt and *Ly49h* transgenic mice was prepared at 36 h after MCMV infection with high viral titer and proteins were quantified by ELISA. Results from 3–6 mice per group are shown. Results shown are representative of one experiment out 2. *P<0.05, **P<0.01, ***P<0.001.

In C57BL/6 inbred strains of mice, the control of MCMV replication occurs via the expression of Ly49H by NK cells. Here, the mechanism underlying this Ly49H-independent susceptibility was studied in the FVB and A/J strains carrying *Ly49h* transgene (A-*Ly49h* and FVB-*Ly49h* [13], respectively). In a steady state situation, Ly49H is expressed by approximately 50% of NK cells derived from B6 mice [13], [18]. Consistent with these data, B6 mice had 50% Ly49H+ NK cells, however Ly49H staining on NK cells derived from FVB-*Ly49h* and A-*Ly49h* mice were both significantly lower than B6 NK cells (Figures 1A and 1B). No differences in the expression of other Ly49 receptors were detected between the FVB-*Ly49h* or A-*Ly49h* strains and their control littermates indicating that the introduction of *Ly49h* gene did not affect the NK cell gene expression repertoire (Figures S1A and S1B). Given that the only known ligand of Ly49H is the viral protein m157, we sought to ensure that this interaction remained intact in A-*Ly49h* mice. We tested the m157-Ig binding capacity and found no strain-dependent differences in m157 binding between B6, FVB-*Ly49h* and A-*Ly49h*. However as expected, m157 binds to the inhibitory Ly49I [19] expressed by FVB NK cells (Figures 1A and 1B). Altogether, these results show that, in A-*Ly49h* and FVB-*Ly49h* mice, Ly49H is properly expressed and can recognize MCMV.

To assess the protective capacity of Ly49H in the A/J (named A in the rest of the manuscript) and FVB backgrounds, we examined several parameters of MCMV infection in these mice. We first challenged the mice with a dose of MCMV that is lethal in the absence of Ly49H expression. As expected, A-WT littermates and B6.*Ly49h^−/−^* mice exhibited drastic weight loss and death compared to B6 and FVB-*Ly49h* mice which survived to the experimental endpoint (Figures 1C and 1D). Remarkably, and similar to the A/J parental control, the A-*Ly49h* mice showed early signs of distress with a precipitous drop in body weight and 100% mortality within the first week of infection (Figures 1C and 1D). To determine whether the observed mortality was a result of unchecked viral replication, we next infected the mice with a low dose of MCMV and assessed liver and spleen viral titers at day 3 post infection. All the mice expressing Ly49H were able to control viral replication efficiently as compared to the mice that lacked the receptor (Figures 1E and 1F). However despite equal expression of Ly49H by NK cells from A-*Ly49h* and FVB-*Ly49h* the viral load in the spleen and liver was significantly increased in A-*Ly49h* mice (Figures 1E and 1F). Viral replication persisted to later time points post infection and was significantly higher in the liver, spleen and heart of A-*Ly49h* at days 3 and 6 compared to other Ly49h expressing mice (B6 and FVB-*Ly49h*). The virus was cleared from all organs by day 10 with the exception of the salivary glands (Figure S2). Finally we interogated whether the early death observed in A-Ly49h mice was the consequence of a systemic increase in inflammatory mediators upon MCMV infection. To this end, we quantified the level of inflammatory proteins at 36 h post MCMV infection in A-*Ly49h* and FVB-*Ly49h* mice as well as their WT counterparts. We found no significant differences between the A-WT and FVB-WT mice in terms of cytokine levels (Figure 1G). However in the Ly49h transgenic mice, the serum levels of all the cytokines tested and the acute phase protein SAA, a marker of tissue injury, was significantly higher in A-Ly49h mice compared to FVB-Ly49h mice (Figure 1H). Thus, the overwhelming inflammation in the A-Ly49h mice might contribute to their early death. Altogether, these results demonstrate that A/J mice are highly susceptible to MCMV infection even with transgenic expression of Ly49H. They further suggest that NK cells from A-*Ly49h* are incapable of efficiently controlling MCMV infection regardless of the amount of viral inoculum.

### Increased viral susceptibility is independent of NK cell proliferation and Gzmb and Prf expression

**Figure 2:**
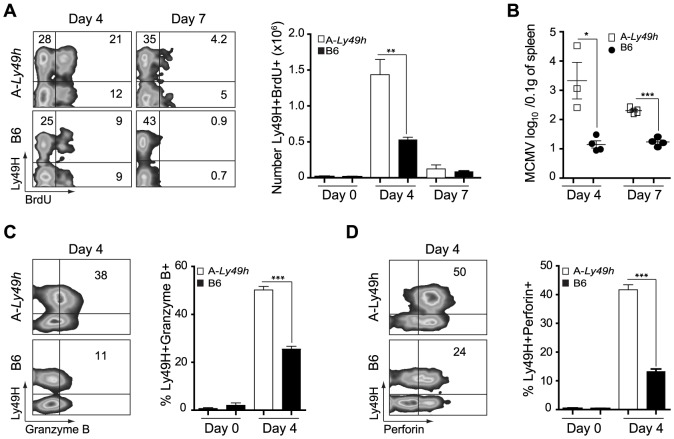
NK cells from A/J mice can proliferate and produce Granzyme B and Perforin following MCMV inoculums. Splenocytes were harvested from naïve B6 and A-*Ly49h* mice (day 0) or mice infected with 2500 PFU MCMV (n = 3 mice/time point). Time points post-infection are indicated in the figure. (A) BrdU incorporation was analyzed on CD3^−^DX5^+^ Ly49H^+^ NK cells by flow cytometry. (B) Spleen viral titers were determined by PA. Intracellular (C) Granzyme (D) Perforin expression was analyzed by flow cytometry on CD3-DX5+ Ly49H+ NK cells. Representative plots from individual mice are shown. The percent of Ly49H+ NK cells positive for Gzmb, and Prf1 are summarized for one experiment. Data were analyzed using two-tailed Student's *t*-test and presented as mean ± SEM and significant *P* values are indicated. *P<0.05, **P<0.01, ***P<0.001. These data are representative of 2–3 independent experiments.

In order for NK cells to proliferate and kill target cells following MCMV infection, their ability to engage activating receptors and to release mature cytotoxic granules such as Gzmb and Prf must be intact. As A-*Ly49h* NK cells could fully recognize m157, we investigated the ability of these cells to proliferate and to become cytolytic upon MCMV challenge. In A-*Ly49h* mice, we observed a significant increase in the number of splenic BrdU^+^ Ly49H^+^ cells at day 4 post-infection, indicative of sustained proliferation (Figure 2A). Cell proliferation was markedly absent in the Ly49H^+^ NK cells from B6 and FVB-*Ly49h* mice (Figures 2A and S3A). Despite a decrease in proliferation in all strains by day 7 post infection, the percentage of BrdU staining remained significantly higher in A-*Ly49h* mice. Since increased Ly49H^+^ NK cell proliferation has been associated with inefficient viral clearance [20], we examined viral replication in the spleen at days 4 and 7 post infection. While B6 and FVB-*Ly49h* mice had largely cleared MCMV from the spleen, viral burden remained significantly high in A-*Ly49h* mice at 4 and 7 days post infection (Figures 2B and S3B). Although it is well established that A/J and B6 NK cells express similar amounts of Gzmb and Prf when stimulated with IL-15 [14], we aimed to rule out the possibility that A-*Ly49h* NK cells are intrinsically incapable of mounting an effective cytotoxic response *in vivo* upon MCMV infection (Figures 2C and 2D). Expression of Gzmb and Prf were assessed at day 4 post infection. We found that Gzmb and Prf expression was drastically increased in Ly49H^+^ and Ly49H^−^ NK cells derived from the A-*Ly49h* mice compared to cells derived from B6 mice and, to a lesser extent, those derived from FVB-*Ly49h* mice (Figure S3D). These results indicate that A-*Ly49h* NK cells, while highly proliferative, are still capable of mounting an effective cytotoxic response in the context of MCMV infection.

### NK cells exhibit an intrinsic defect in IFNγ production

**Figure 3:**
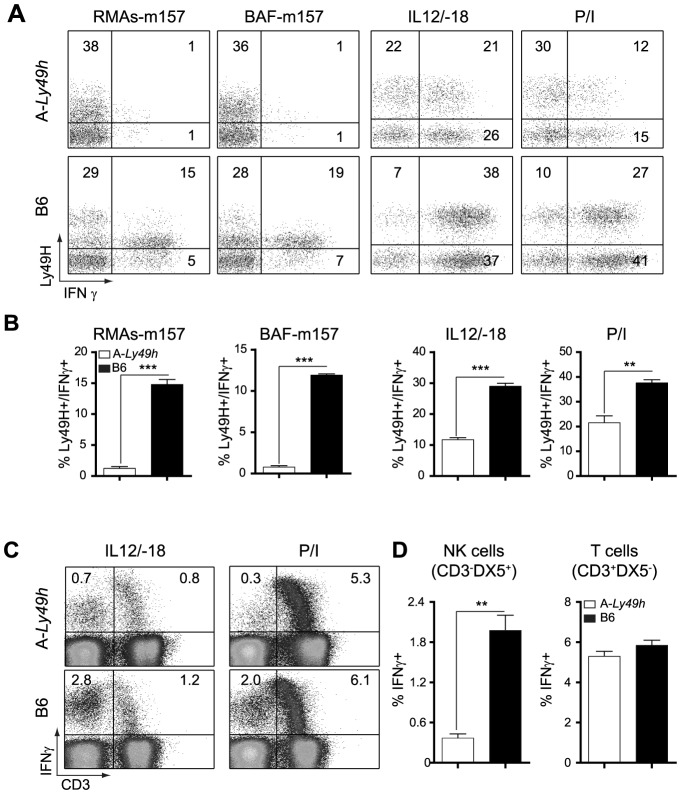
Decreased IFNγ production by Ly49H^+^ NK cells in A/J mice. Splenocytes from B6 and A-*Ly49h* mice were harvested and stimulated with RMAs-m157, BAF-m157, IL12/IL18 or PMA and ionomycin (P/I) for 3–5 h. (A) Representative dot plots demonstrating IFNγ production following stimulation and gaited on CD3^−^DX5^+^ Ly49H^+^ NK cells. The numbers represent the percentage of Ly49H^+^ producing IFNγ. (B) Graphical representation of IFNγ production by CD3^−^DX5^+^ Ly49H^+^ NK cells following stimulation. (C) Representative dot plots showing IFNγ production following stimulation and gaited on CD3^+^ or CD3^−^ cells. Numbers represent the percentage of cells producing IFNγ. (D) Graphical representation of IFNγ production by CD3^−^ DX5^+^ (T cells) or CD3^+^ DX5^−^ (NK cells) after P/I stimulation. Data were analyzed using two-tailed Student's *t*-test and presented as mean ± SEM and *P* values of significant results between groups are indicated. *P<0.05, **P<0.01, ***P<0.001.

It is well establish that IFNγ is required for protection against acute MCMV infection [17], [21]. We therefore investigated the ability of A-*Ly49h* NK cells to produce IFNγ upon stimulation. NK cells from A-*Ly49h* and B6 mice were exposed to m157-target cells by co-culturing spleen cells with RMAs-m157. These cells lack MHC-I expression and, as such, the triggering of Ly49H can be assessed in the absence of inhibitory signals originated from MHC-I/Ly49 receptor interactions. B6 Ly49H^+^ NK cells robustly produced IFNγ after 4–6 h of stimulation with their m157-target cells, whereas A-*Ly49h* NK cells lacked IFNγ^+^ cells in the same conditions (Figure 3A). Conversely, to address the effect of MHC-I expression we co-cultured A-*Ly49h* splenocytes with BAF-m157, cells derived from BALB/c bone marrows and possessing an H-2^d^ haplotype. We found that the presence of MHC-I did not change the ability of A-*Ly49h* NK cells to produce IFNγ. To determine whether this defect was pathway specific, we interrogated whether these NK cells could produce IFNγ if stimulated through the IL12/IL18 receptors. Interestingly, B6 Ly49H+ NK cells produced twice as much IFNγ as Ly49H+ NK cells from A-*Ly49h* mice. Finally, to bypass receptor proximal signaling we activated the NK cells with phorbol myristate acetate (PMA) and the calcium ionophore ionomycin (Iono) that stimulate the cells directly by mobilizing free calcium ions and activating PKC enzymes. The differential IFNγ production was even more striking in cells stimulated with PMA plus Ionomycin (P/I) (Figures 3A and 3B). IFNγ production by FVB-*Ly49h* Ly49H^+^ NK cells closely mirrored those observed in B6 NK cells. Overall, the production of IFNγ by NK cells from A-*Ly49h* was significantly decreased when compared to B6 NK cells (Figure 3C and 3D). Determination of TNFα showed similar levels in NK cells from A-WT or B6 mouse strains. As stimulation of T cells by IL-12/IL18 or P/I also results in the production of IFNγ, we sought to determine whether the defect in IFNγ production in A-*Ly49h* was NK cell specific. Remarkably, T cells from A-*Ly49h* mice were found to produce similar amounts of IFNγ as those from B6 mice (Figure 3D). Collectively, these data suggest that A mice carry a genetic defect that specifically affects the production of IFNγ by NK cells and that functions downstream of several immune receptor (cytokine, PRR, NKR) pathways or chemical stimulation.

### Deficiency in IFNγ production by NK cells is independent of the NKC or the MHC

**Figure 4:**
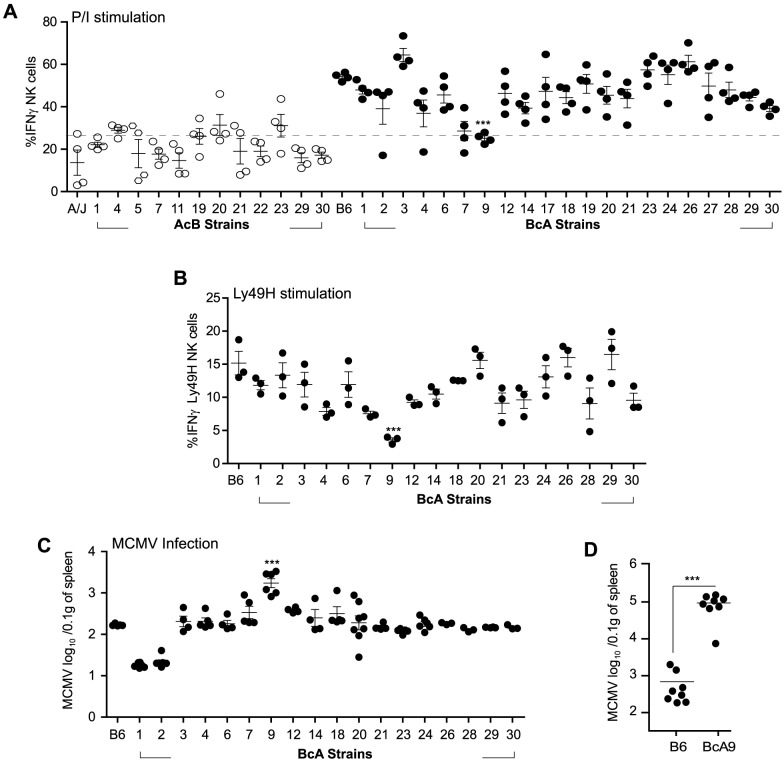
Decreased IFNγ production by NK cells in A-*Ly49h* mice occurs independently of the NKC and H2 and is linked to MCMV susceptibility. (A) Freshly isolated splenocytes from 33 RCS strains and parental strains were stimulated with P/I for 4 h. The percentage of CD3^−^DX5^+^ NK cells expressing intracellular IFNγ is shown and represents data from three pooled experiments. Data were analyzed using a two-way ANOVA. (B) Splenocytes from 18 BcA strains expressing Ly49H receptor were co-cultured with RMAs-m157 for 3 h. Intracellular IFNγ was analyzed on CD3^−^DX5^+^ Ly49H^+^ NK cells by flow cytometry. Data were analyzed using a two way ANOVA with a bonferroni post test. (C) 18 BcA strains expressing Ly49H receptor and the parental B6 strains were infected with low dose of MCMV. Spleen viral titer was quantified 3 days later by PA. Data were analyzed using a two way ANOVA with a bonferroni post test. (D) B6 and BcA9 mice were infected with high dose of MCMV. Spleen viral load was quantified at day 3 p.i. Data were analyzed using two-tailed Student's *t*-test and presented as mean ± SEM and *P* values of significant results between groups are indicated. (A, B,C and D) Statistical differences between B6 and BcA9 strains are shown *P<0.05, **P<0.01, ***P<0.001.

NK cell responsiveness is dictated by both the NK cell repertoire and the interaction of these with their cognate MHC-Class I molecules [22]. To evaluate whether one of these variables affected the fate of A-*Ly49h* NK cells, we used a panel of AcB/BcA recombinant congenic strains (RCS) in which each AcB strain inherits ∼12.5% genes from the B6 genome and ∼87.5% genes from A/J, and reciprocally for each BcA strain. We screened the RCS panel for two parameters of NK cell responsiveness: IFNγ production and control of viral replication. Splenocytes from all strains were stimulated with P/I for 4 h and intracellular IFNγ was determined by FACS in CD3^−^DX5^+^ NK cells (Figure 4A).

Surprisingly, the IFNγ production profile clearly distinguished the A/J from the B6 background. Production of IFNγ was much lower in AcB NK cells (below a 25% threshold) than in BcA NK cells and was not dependent on the inheritance of the A/J NKC or H2 (as evidenced by the elevated production of IFNγ in BcA17, BcA19, BcA27, and BcA4 strains) (Figure 4A and Table S1). We found that two strains, BcA7 and BcA9 showed decreased on IFNγ production similar to the AcB strains, however this decrease was much more profound in the BcA9 strain (Figure 4A). This genetic background-dependent difference in IFNγ production, however, was completely absent in T cells from the same strains (Figure S4A). To determine whether the differences observed were maintained upon triggering of Ly49H receptor, splenocytes from the BcA strains (with the exception of BCA17, 19 and 27 which inherited the NKC from the A strain and do not express Ly49H) were co-cultured with RMAs-m157 and IFNγ production by NK cells was analyzed by FACS. The decreased IFNγ expression characteristic of A-*Ly49h* NK cells was only observed in BcA9 NK cells (Figure 4B). To assess the impact of differential IFNγ production on MCMV replication, we investigated viral titers in the spleens of BcA mice 3 days post infection. The AcB strains were excluded as none of them had inherited the B6 NKC (Table S1). As expected, Ly49H expression was required for efficient control of viral replication; high viral loads were observed in strains possessing the A/J NKC: A/J, BcA17, BcA19, and BcA27. Among the BcA strains possessing the B6 NKC, increased susceptibility to MCMV infection was only observed in the BcA9 strain (Figure 4C). This susceptibility was also confirmed when B6 and BcA9 were infected with high MCMV doses (Figure 4D). Given that BcA9 retains 12.5% of the A background, we then investigated whether NK cells from B6 and BcA9 behave similarly in terms of expression of NK cell receptors and function. We found that CD11b, Ly49G. Ly49HIC and NKG2A are similarly expressed between the two strains with a slight decrease of KLRG1 (in BcA9), (Figure S5A). In addition, NK cells from both strains were capable of rejecting MHC-I deficient cells and of killing m157 expressing splenocytes efficiently (Figure S5B).

Collectively, the data indicate that A-*Ly49h* and BcA9 NK cells behave similarly with regard to viral control and IFNγ production. Moreover, the NK cell-specific, strain-dependent differential production of IFNγ suggested that this phenotype is genetically determined.

### The defect of IFNγ production by NK cells is linked to a single locus on chromosome 10

**Figure 5:**
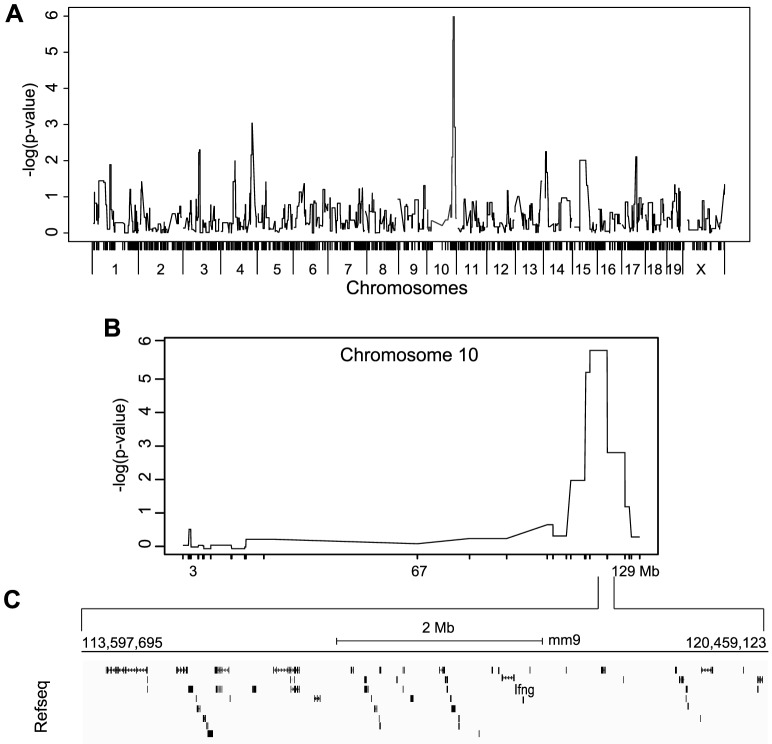
Mapping of IFNγ production by NK cells reveals a single locus on chromosome 10. (A) Genome-wide linkage analysis was done using mice from the 33 RCS strains outlined in Figure 4A. IFNγ production by NK cells following P/I treatment was used as the mapping trait. The negative log genome-wide *p* values are shown. (B) Chr 10 negative log genome-wide *p* values of IFNγ production by NK cells upon P/I treatment. (C) Map of the 6.6 Mbp relevant interval in chr 10 harboring 45 genes in black rectangles (adapted from UCSC mouse genome browser, mm9).

To map the genetic determinants underlying IFNγ production by NK cells, we performed a genome wide association analysis of IFNγ production as a quantitative trait using efficient mixed model association (EMMA) [23]. 1215 SNPs overlapping the relevant break points and representing the genetic diversity of the RCS strains were used to map a single quantitative trait locus (QTL) on chromosome 10 (113.59–120.45, p<10^−8^). This locus was associated with IFNγ production by NK cells (Figure 5A). The same analysis performed using IFNγ production by T cells did not show any significant QTLs (Figure S4B). The physical size of the chromosome 10 target interval was 6.6 Mbp containing 39 Refseq annotated genes including *Ifng* (Figure 5C and Table S2). To narrow the pool of candidates, we compared exome sequencing data from A and BcA9 strains against the reference B6 genome sequence with a required minimum of 10× sequencing depth coverage. 30 non-synonymous coding polymorphisms were identified in 11 genes in both the A and BcA9 strains (Table S3). We then removed variants found in public databases (http://www.sanger.ac.uk/cgi-bin/modelorgs/mousegenomes/snps.pl, http://www.informatics.jax.org/and http://cgd.jax.org/cgdsnpdb/) shared between A and C3H, DBA/2 or 129S1 strains, as NK cells from these strains do not show a defect in IFNγ production (Table S3 and Figure S6). This left 11 coding variations affecting 8 genes mapping mostly to the 3′ and 5′UTRs. Given that the defect in IFNγ expression was not observed in T cells, we hypothesized that the underlying gene had to be expressed exclusively in NK cells. Thus, for the remaining 11 variations, we performed an *in silico* analysis of gene expression using public databases [24] (https://www.immgen.org/and https://www.biogps.org) [25]. We found that none of the 8 candidate genes were NK cells specific, thus they were given lower priority for further study (Table S3). Collectively, our mapping strategy and enquiry of the genetic variation underlying candidate genes within the target interval indicated that the NK cell intrinsic defect in IFNγ production is linked to chr. 10 and is unlikely to be due to a protein-coding defect.

### IFNγ production defect by NK cells is determined at the transcriptional level and linked to increased susceptibility to MCMV infection

**Figure 6:**
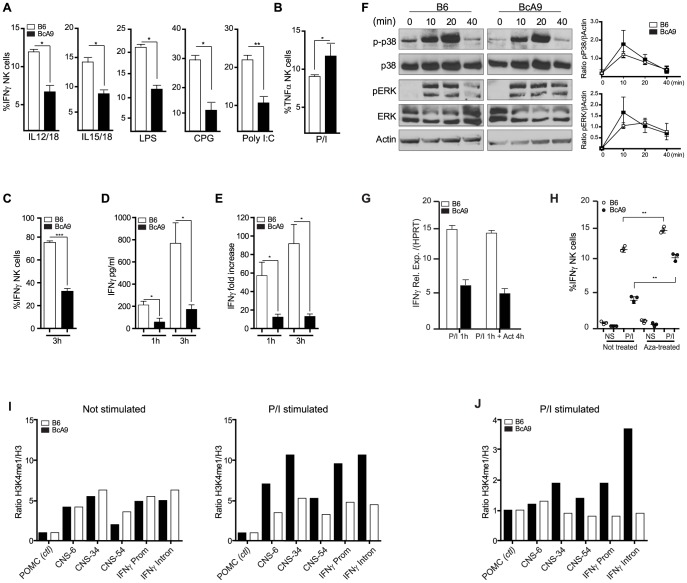
Decreased IFNγ production by BcA9 NK cells is independent of impaired MAP kinase pathway signaling or RNA instability. (A) B6 and BcA9 splenocytes were stimulated with indicated Pathogen-associated molecular patterns for 16–18 h and IFNγ in NK cells was quantified intracellularly, 3 mice per group were used. (B), NK cells from indicated strains were stimulated with P/I and intracellular TNFα was analyse by FACS. (C, D and E) NK cells from indicated strains were generated by culturing splenocytes in IL-2 media for 6 days. (C) NK cells were stimulated for 3 h with P/I and intracellular IFNγ was analyzed by flow cytometry. (D) IFNγ production was quantified from the supernatants at the indicated times (E) Expression levels of IFNγ were determined by qPCR at the indicated times. Data represent means ± SEM of triplicates. Sample data were analyzed using two-tailed Student's *t*-test and presented as mean ± SEM and significant *P* values are indicated. *P<0.05, ***P<0.001. Similar results were obtained in three independent experiments. (F) Western blot analyses p-p38, p38, ERK, pERK, and actin were performed with equal protein loading of each sample. Quantification of p-P38 and pERK against βActin are shown. (G) B6 and BcA9 NK cells were cultured in rIL-2 and stimulated for 1 h with P/I. Cells were treated or not with actinomycin D (Act) for 4 h and IFNγ transcript was quantified by qPCR. Results of 3 mice per group are shown. (H) B6 and BcA9 NK cells were cultured 6 days, and treated or not with 5-aza-2-deoxycytidine (Aza). Cells were then stimulated or not with P/I for 1 h and IFNγ was analysed by FACS as described before. Results of 3 mice per group are shown. Similar results were obtained in another independent experiment. **P<0.01. NK cells from indicated strains were generated by culturing splenocytes in IL-2 media for 6 days. (I) L-2 derived NK cells were left none stimulated or stimulated for 1 h with P/I subjected to ChIP against H3 and H3K4me1. Levels of the H3K4me1 active chromatin mark were measure relative to levels of H3 for known *Ifnγ* regulatory regions. (J) Fresh NK cells were stimulated for 1 h with P/I subjected to ChIP against H3 and H3K4me1 and level of H3K4me1 active chromatin mark was measured as before.

Because the impaired IFNγ production was observed after P/I treatment in total NK cells from the BcA9 strain, we questioned whether this decrease of IFNγ production would be observed with the treatment of other stimuli as for A-*Ly49h* mice. We found that decreased IFNγ production by BcA9 NK cells was also observed when total splenocytes where incubated with IL-12/IL-18, IL-15/IL-18 as well as LPS, CPG, and Poly I:C (Figure 6A). As for the expression of TNFα in B6 and A strains, we found no decrease TNFα production by NK cells from BcA9 in comparison with B6 mice after P/I stimulation (Figure 6B). To better characterize the mechanism underlying differential IFNγ regulation, IFNγ mRNA and protein levels were measured in NK cells from B6 and BcA9 mice. We first generated NK cells by co-culturing splenocytes with IL-2 for 6 days. B6 and BcA9 LAK cells were then stimulated with P/I for 1–3 hours, after which IFNγ expression was determined. Intracellular and secreted IFNγ levels were decreased in BcA9 cells compared to B6 (Figures 6C and 6D). IFNγ mRNA was also decreased in BcA9 cells indicating that the differential regulation occurs at the level of transcription (Figure 6E). As the *Ifng* gene itself was located within the QTL, we looked for mutations in both the promoter region and the well-characterized regulatory elements required for proper IFNγ expression and as expected since IFNγ production is not affected in T cells, none were identified.

Stimulation with P/I activates the PKC pathway and increases the calcium flux which in turn induces the phosphorylation of Erk and P38, events that are crucial for the transcriptional regulation of IFNγ [26]. We found that IFNγ production was not effected in T cells after P/I stimulation (Figures 3C, D and S4). We therefore asked whether this pathway was also functional in NK Cells, by determining whether NK cells from BcA9 mice had impaired signalling following P/I stimulation. However, no differences in Erk or P38 phosphorylation were observed (Figures 6F). These data indicate that the dysregulation of IFNγ is not part of any upstream signalling or transcriptional pathway or of any known IFNγ cis-regulatory element, yet the dysregulation is manifested at the level of transcriptional control.

To investigate whether the decreased IFNγ production in the BcA9 mice was a consequence of mRNA stability, we treated NK cells from B6 and BcA9 mice with the transcriptional inhibitor actinomycin D after P/I stimulation and we found no difference in RNA levels between treated and non treated NK cells in both strains (Figure 6G). These data suggest that mRNA degradation in NK cells from the BcA9 strain is not involved in the decreased production of IFNγ. We then examined if differences in DNA methylation might explain the decreased IFNγ production in NK cells from BcA9 mice by treating NK cells with the DNA methylase inhibitor 5-aza-2-Dexoxycytidine (Aza). We found that IFNγ production was increased in both strains after Aza treatment. However the magnitude of increase was higher in NK cells from BcA9 (2,5 times) than B6 mice (1.3 time) (Figure 6H). These data suggest that DNA methylation is somehow involved in the genetic regulation of IFNγ production in NK cells from BcA9 mice. We finally performed chromatin immunoprecipitation (ChIP) assays to determine whether BcA9 and B6 NK cells possess different epigenetic marks in the *Ifnγ* locus. Monomethylation of H3 lysine 4 (H3K4me1) is a histone mark associated with cis-regulatory elements. This mark is enriched at the *Infg* promoter and at 4 upstream CNSs (−6 kb, −22 kb, −34 kb, and −54 kb) during *Ifnγ* transcription in both NK and T cells. We found that the enrichment of H3K4me1 in non-stimulated IL-2 derived NK cells was similar between B6 and BcA9 mice (Figure 6 I left). This might indicate that the decreased *Ifnγ* transcription observed in BcA9 NK cells is not a consequence of *Ifnγ* long term locus silencing. However, following 1 hour of P/I treatment, increased enrichment of the H3K4me1 was observed at the *Ifnγ* promoter and CNSs of B6 NK cells but not of BcA9 NK cells (Figure 6 I right). In addition, in another set of experiments done on fresh NK cells, the results correlate with those outlined above (Figure 6 J).These results suggest that chromatin remodelling involved in P/I induced *Ifnγ* transcriptional activation is altered in NK cells derived from BcA9 mice.

**Figure 7:**
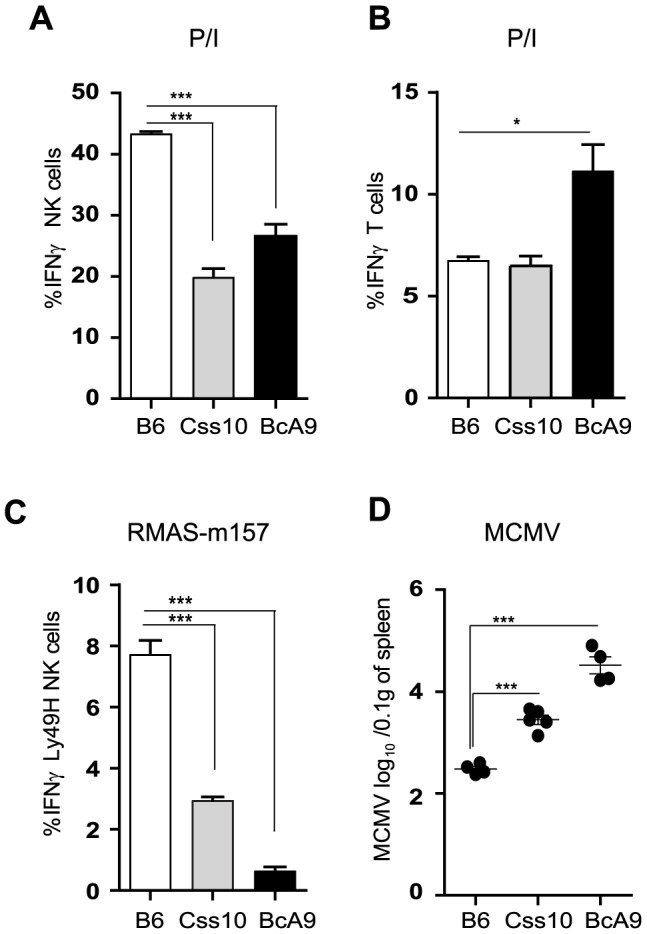
Chromosome 10 controls both IFNγ production and MCMV resistance. (A, B) Freshly isolated splenocytes from B6, Css10 and BcA9 mice were stimulated with P/I for 4 h. The plots are gaited (A) on CD3^−^DX5^+^ NK cells (B) CD3^+^DX5^−^ T cells and the percentage of cells expressing intracellular IFNγ is shown. (C) Splenocytes from indicated strains were incubated for 4 h with RMAS-m157 and the percentage of Ly49H^+^ NK cells expressing intracellular IFNγ is shown. Data were analyzed using one way ANOVA with Bonferoni post-test and presented as mean ± SEM. **P<0.01, ***P<0.001. (D) Indicated mice were infected with MCMV and viral load was quantified from the spleen at day 3 p.i. Data were analyzed using two-tailed Student's *t*-test and presented as mean ± SEM. ***P<0.001. Similar results were obtained in another independent experiment.

To confirm our linkage analysis and to rule out that the genetic alteration occurred elsewhere in the genome and does not act in *trans* on *Ifng* gene, we assessed IFNγ production in Css10 mice. This strain possesses chromosome 10 from A mice on a B6 background. Consistent with an A strain specific effect, production of IFNγ by Css10 and BcA9 NK cells was significantly decreased after stimulation with P/I or triggering of Ly49H receptor (Figure 7A, 7C). Once again, IFNγ production by T cells was not affected, confirming that the locus on chromosome 10 controls only IFNγ production in NK cells (Figure 7B). To address whether the decreased IFNγ production in these mice was likewise linked to MCMV susceptibility, B6, Css10 and BcA9 mice were infected with MCMV. Although they showed lower MCMV load than the BcA9 strain, Css10 mice were significantly more susceptible to MCMV infection than B6 mice as determined by viral titers in spleen (Figure 7D). We also investigated whether the surface expression of NK cells receptors was differently expressed between strains, before and after MCMV infection. We found that after infection, the level of NK cell receptor was similarly expressed between B6, BcA9 and Css10 mice (Figure S7). These data indicate that the chromosome 10 locus controls both IFNγ expression by NK cells and subsequent susceptibility to MCMV infection.

## Discussion

We report that deficiency in IFNγ production by NK cells upon Ly49H/m157 engagement is associated with increased susceptibility to MCMV infection independent of NK cells capability to release cytolytic granules. This defect is not only linked to NK cell stimulation through an activating receptor, but seems to affect various signaling pathways for IFNγ secretion. Decreasing IFNγ secretion is seen at the level of IFNγ mRNA transcription upon engagement of ITAM-dependent and ITAM-independent upstream signaling events. We used the AcB/BcA panel of recombinant congenic strains to map the regulation of IFNγ production to a 6.6 Mbp region on chromosome 10. No NK cell specific deleterious protein-coding mutations were identified in that interval however we confirmed that the locus controls both IFNγ production and viral spread.

The importance of IFNγ in the modulation of MCMV infection stems from several studies using IFNγ and IFNγ receptor deficient mice. Although some reports using mice of mixed 129/B6 background suggested that the production of IFNγ by NK cells may be a predominant antiviral mechanism in the liver at six days post-infection [21], [27], newer studies using mice of uniform C57BL/6 origin showed that IFNγ is required for the control of MCMV in the spleen and liver as early as three days post-infection [17]. We note, however, that CSS10 mice showed about ten-fold lower viral replication than BcA9 animals suggesting that this strain carries additional MCMV-susceptibility loci, the mapping of which would require secondary crosses using BcA9. Regardless of these considerations, our QTL analysis and co-occurrence of defective antiviral function in CSS10 mice indicate that a specific decrease in IFNγ production by NK cells and not by T cells is associated with increased MCMV susceptibility *in vivo*.

NK cell hyporesponsiveness is often associated with self-tolerance. Self-tolerance occurs in NK cells that develop in MHC Class I deficient hosts or that lack self MHC-I-specific inhibitory receptors [6], [7], [9]. In these cases, impaired killing and IFNγ production is observed in NK cells following engagement of activating receptors. It follows that functional competence requires the interaction of MHC-I molecules with their cognate inhibitory receptors during NK cell development. As A-*Ly49h* mice exhibit impaired IFNγ production compared to B6 counterparts, it is possible that NK cells from these strains are differentially licensed by their respective MHC-I molecules. Interestingly, hyporesponsive NK cells from MHC-I deficient mice produce normal IFNγ upon stimulation with P/I and can clear MCMV infection similarly to WT NK cells [6], [9], [28], [29]. We show that BcA9 NK cells are hyporesponsive despite carrying a B6 MHC-I region. Additionally, we show that decreased IFNγ production was sustained upon P/I stimulation and MCMV replication was increased compared to WT counterparts. These indicate that the defect in IFNγ production does not result from increased NK cell self-tolerance. Altogether, these findings make the interaction between MHC-I and the NK-cell receptor repertoire an improbable player in our NK cell hyporesponsiveness model.

NK and T cells use redundant, non overlapping signaling pathways for IFNγ production. Such pathways regulate IFNγ production through a number of transcription factors including T-bet [30], STAT4 [31], STAT5 [32], [33], IκBζ [34], NF-kB family members [35], and Runx3 [36]. Here we show that decreased expression of IFNγ is specific to NK cells suggesting that a defect in any gene encoding a master transcription factor for IFNγ expression is unlikely. In addition, our genetic analysis ruled out any coding polymorphisms in these transcription factors as being linked to low IFNγ expression. Finally, as the I IFNγ defect is seen in both the BcA9 and CSS10 strains, it is unlikely that it is controlled by a de novo mutation located outside of our locus of interest.

Phorbol esters such as PMA in addition to Ionomycin are potent activators of IFNγ production in NK cells and T cells. The pathways involve the activation of the PKC isozymes (α, β Ι, β ΙΙ, γ, δ, ε, θ) which in turn activate MAPK kinases such as ERK, p38 and JNK. Engagement of NK cell activating receptors that signal through ITAMs also results in prompt activation of PKC θ which is essential to induce IFNγ production [26]. Consistent with this idea, we observed that the MAPK pathway was not implicated in impaired IFNγ production in NK cells from the BcA9 strain. More importantly, we found that defect of IFNγ production by NK cells was correlated with changes in IFNγ mRNA levels independently of decreased mRNA stability. These data suggest that the dysregulation of IFNγ production is not modulated by post-transcriptional or translational events.

Our results demonstrate that the defect in IFNγ production relates to decreased expression of IFNγ mRNA in NK cells suggesting a cis-regulatory mechanism. In fact, we found that differences in DNA methylation might in part explain the defect in IFNγ production. Proper transcription of IFNγ is dependent on the binding of specific transcription factors to conserved non-coding sequences (CNSs) surrounding the *Ifng* gene. To date, nine CNSs have been identified within ∼120 kb flanking the murine *Ifng* locus [37]–[40]. These become selectively activated in differentiated cells that express IFNγ [41]. Recent studies using BAC transgenic mice harboring various deletions both upstream and downstream of the human *IFNγ* gene revealed that distal CNSs have cell type-specific function [42], [43]. In this regard, our *in silico* attempt to identify mutations that might abrogate the binding of transcription factors, whether in the promoter or CNSs of A mice, did not identify any deleterious modifications. In a recent study, NeST, a lncRNA located within our *Ifng* locus has been shown to be expressed in T cells and selectively affects IFNγ expression on CD8^+^ T cells [44]. By analyzing public databases we identified only 3 ancestral polymorphisms within NeST between B6 and A mice (rs29312535, rs29330422 and rs49496559). Two of these were discounted because they were also present in the C3H and 129S1 mouse strains, which do not exhibit IFNγ expression deficits. It has been shown that the primary mechanism of action for polymorphism differences in NeST in T cells was to affect its own RNA expression levels, which lead to modulated IFNγ production [44]. Thus, the remaining SNP (rs29312535) is unlikely to be involved in the decreased IFNγ production by NK cells in A mice since no difference in the expression of NeST in NK cells from BcA9 and B6 strains was found. However, we cannot exclude that the SNP (rs29312535) might alter the physical interaction of NeST with IFNγ transcriptional complex in NK cells and this possibility has to be explored in future work. Interestingly, a recent genome-wide ChIP analysis in the *Ifng* locus of active (H3K4me2) and repressive (H3K27me3) chromatin marks from Th1 and Th17 γδT cell subsets revealed additional marks for putative active regulatory elements (Figure S8) [45]. Thus, active transcriptional marks were found upstream and downstream of the *Ifng* gene, six of which were directly located within the *NeST* region (Figure S8). The presence of these new active transcriptional marks in the vicinity of *Ifng* suggests that our understanding of IFNγ production by T cells and more specifically by NK cells needs further investigation. In addition, proper studies using ChIP-Seq to evaluate activating and repressive epigenetic modifications will provide a more comprehensive view of IFNγ regulation by NK cells.

Specific expression of a number of cytokines, including IFNγ, IL4, IL13 and IL17 have been shown to be tightly regulated by extremely well conserved *cis*-regulatory elements [46]. Deletion of these elements has been correlated with decreased cytokine production, thereby shedding light on the molecular basis for lineage specific-transcription. Most studies evaluating the control of IFNγ expression have focused on CD4^+^ T cell differentiation into Th1 or Th2 cells. Th1 cells express IFNγ but not IL4, IL13 and IL5 whereas the opposite is true in Th2 cells [41]. The underlying mechanism responsible for this differentiation involves the repression of the *Ifng* locus in Th2 cells and the *Il4* and *Il13* loci in Th1 cells [47], [48]. Thus specific epigenetic events determine chromatin permissiveness and accessibility of genes for the transcriptional machinery. In contrast to T cells, the *Ifng* locus in NK cells is not repressed, which might allow rapid response to external stimuli [31]. However, the specific mechanisms providing lineage specific *Ifng* transcription are still not well understood. To conclude, the subtle quantitative allelic differences found in animal models have more often revealed that equivalent polymorphisms exist in humans. Our analysis of the mechanism of viral control and IFNγ production by NK cells have led us point out the existence of “modulatory” elements that dictate the production of IFNγ by NK cells. These findings suggest that deeper knowledge of IFNγ regulation by NK cells will offer insight into clinical immune pathologies and direct strategies for intervention.

## Materials and Methods

### Ethics statement

All mice were kept under specific pathogen free conditions and handled according to the guidelines and regulations of the Canadian Council on Animal Care. Mice experimentation protocol (Protocol number 4791) was approved by the McGill Facility Animal Care Committee (FACC).

### Animals

C57BL/6 (B6), A/J (A in the text), and C57BL/6J-Chr 10^A/J^/NaJ (Css10) were purchased from The Jackson (Jax, Bar Harbor, ME) and FVB/N were purchased from Charles River Laboratories (Wilmington, MA). FVB.*Ly49h* transgenic (FVB.*Ly49h*) mice and B6.*Ly49h^−/−^* mice were obtained as previously described [12], [13]. A.*Ly49h* mice were generated by backcrossing the FVB.*Ly49h* mice onto an A/J WT background for a minimum of 10 generations. A genome scan using 1449 single nucleotide polymorphism showed a 100% similarity between A/J and A.*Ly49h* mice. Transgenic mice were identified by PCR using the *Ly49h* specific marker, D6Ott11 as described previously [12]. Recombinant congenic mice of the AcB/BcA set were derived from two successive backcrosses (N_3_) to either A/J (AcB) or B6 (BcA) parental mice, as previously described [49]. The B6 mice deficient for *H2*-*D^b^K^b^* (B6.*H2* ^−/−^) were kindly provided by Dr. Hidde L. Ploegh (Cambridge, Massachusetts). The m157-Transgenic mouse was kindly provided by Dr. Sandeep K. Tripathy, (Washington University School of Medicine, St. Louis). Mice were bred and maintained in a specific pathogen-free animal facility at McGill University. All experimental protocols were developed in accordance with institutional guidelines of the Canadian Council on Animal Care.

### Virus and infection

Stock salivary gland Virus (SVG) was prepared by passaging the MCMV (Smith strain ATCC VR-1399, lot 1698918) twice in BALB/c mice. Virus was prepared from the homogenate of salivary glands at day 21 post-infection. Viral titer was evaluated in vitro by standard plaque assays (PA) on a confluent CD1 MEF monolayer as previously described [50]. Mice between 7 and 9 weeks were infected intraperitoneally (IP) for the indicated times and doses. To determine the viral titer from target organs, hearts and lungs were perfused with PBS prior to homogenization and MCMV titer was quantified by standard plaque assay.

### Flow cytometry and FACS analysis

FACS was conducted on splenocytes from MCMV-infected and uninfected mice. Single spleen cell suspensions were prepared by grinding the spleen against a 70 µm nylon mesh, lysing red blood cells using ACK lysis buffer and incubating the remaining cells with 2.4G2 antibody to block Fc receptors. Fluorescently labeled antibodies and reagents were purchased from BD Biosciences, eBioscience, BioLegend and R&D Biosystems. For determination of the Ly49H-m157 binding, cells were stained with the m157-Ig fusion protein (gift of L. Lanier, USCF) followed by detection with PE-conjugated goat anti-human IgG1 (Jackson ImmunoResearch Laboratories). Flow cytometry analyses of cells were performed on a FACSCalibur and Canto II cytometer (BD Biosciences) equipped with FACSDiva software and data were analyzed using FlowJo software (Tree Star).

### IFNγ quantification

IFNγ was measured directly from fresh NK cells *ex vivo* or cultured in recombinant rhIL-2 (1000 U/mL) for 6 to 8 days. To analyze intracellular IFNγ, single spleen cell suspensions were plated at 5*10^6^ cells per 6-well plate and co-cultured with 2×10^5^ cells of BAF-m157 or RMA/s-m157 (Gift from L. Lanier, UCSF) per well in 6-well plates. Alternatively, cells were stimulated with PMA (100 ηg/ml)/ionomycin (1 µg/ml), rmIL-12 (10ηg/ml), rmIL-18 (50 ηg/ml) and rmIL-15 (50 ηg/ml) for 5–6 h at 37°C or LPS (5 µg/ml), CPG (5 µg/ml) and PolyI:C (50 µg/ml) for 16 h. In the last 5 h of the incubation time, a 500-fold dilution of GolgiPlug (BD Biosciences) was added. After extracellular staining, cells were permeabilized using BD Cytofix/Cytoperm solution and stained in BD Permwash using anti-IFNγ antibody.

ELISA for IFNγ was quantified from supernatants and performed according to the manufacturer's instructions (eBioscience).

IFNγ transcripts were analyzed by QPCR using HPRT as a control with the following primers; IFNγ: For 5′ cacggcacagtcattgaaag 3′ and Rev 5′catccttttgccagttcctc3′; HPRT: For 5′ggactgattgacaggaggactg 3′ and Rev 5′ggactgattatggacaggactg 3′.

### Enzyme-linked immunosorbent assay (ELISA)

To obtain serum for cytokine and amyloid A quantification, blood samples were obtained by cardiac puncture from mice infected for 36 h with 5000 PFU of MCMV. Serum was isolated by centrifugation for 20 minutes at 3,000 rpm and stored at −80°C. ELISA for IFN-γ and IL-12 p70 (eBioscience), TNFα (Biosource), IFNβ (PBL), SAA (Invitrogen) were performed according to the manufacturer's instructions. For quantification of mouse IFN-α, serum samples were dispensed on 96-well microtiter plates coated with a rat anti–mouse IFN-α antibody (RMMA-1; PBL Biomedical Laboratories). Subsequently, IFN-α was detected with a polyclonal rabbit anti–mouse IFN-α antibody (PBL Biomedical Laboratories) and a donkey anti–rabbit IgG conjugated to horseradish peroxidase (GE Healthcare) as previously described [51]. Absorbance was measured at 450 nm using a Fluostar optima (BMG LabTech).

### Genotyping and mapping

The recombinant congenic strains of mice were genotyped using 1215 markers spanning the entire mouse genome and covering the relevant break points in the AcB/BcA panel of mice [52]. Statistical analyses were performed using the freely available package R. We used a mixed model statistical test designed to correct for genetic relatedness in mouse models known as Efficient Mixed Model Association (http://mouse.cs.ucla.edu/emma/). Negative log p-values are presented for each marker.

### Exome sequencing

Exome capture was performed in A/J and BcA9 Samples using a SureSelect Mouse All Exon kit (Agilent Technologies, USA) and parallel sequencing on an Illumina HiSeq 2000 (100-bp paired-end reads). Reads were aligned to mm9 genome assembly using BWA. Coverage was assessed using BED Tools and showed an average of 58.9× base coverage. Single nucleotide variants (SNVs) and short insertions and deletions (indels) were called using SAMtoolspileup and varFilter with the base alignment quality (BAQ) adjustment disabled, and were then quality filtered to have at least 20% of reads supporting the variant call. Variants were annotated using both Annovar and custom scripts to identify whether they affected protein coding sequence.

### Western immunoblots

B6 and BcA9 NK cells were cultured 5 to 8 days in rIL-2 (1000 U/mL). NK cells were stimulated with P/I for the indicated times. Proteins from cell lysates were separated by standard SDS-PAGE and analyzed by immunoblotting with antibodies specific to p38, phosphorylated p38, ERK1/2, and phosphorylated ERK1/2 (all from Cell Signaling Technology, Danvers, MA). Anti-actin was purchased from Santa Cruz Biotechnology (Santa Cruz, CA) and used as loading control. Densitometry results were analyzed with Image J software.

### NK cell treatments

For the RNA stability analysis, Actinomycin D (Sigma) treated B6 and BcA9 NK cells were cultured for 5 to 8 days in rIL-2 (1000 U/mL) and stimulated for 1 h with P/I. Cells were washed with complete RPMI, then left untreated or treated with 10 µg/ml of actinomycin D for 4 h at 37°C. The RNA was then extracted as previously described and IFNγ transcript was analyzed by qPCR. For the analysis of DNA methylation, B6 and BcA9 NK cells were cultured for 6 days and treated or not with the methyltransferase inhibitor 5-aza-2-deoxycytidine (Sigma) at 10 µM/ml daily in the presence of rIL-2 for 72 h. Cells were then stimulated or not with P/I for 1 h and IFNγ was analysed by FACS as described before.

### Chromatin immunoprecipitation

NK cells cultured in recombinant rhIL-2 (1000 U/mL) for 6 to 8 days or highly enriched T cells (sorted using magnetic cell sorting) (MACS; Miltenyi) were prepared from B6 and BcA9 mice. 10 million cells were plated on 100 mm culture grade Petri dishes. The next day, culture medium was changed and cells were treated with a vehicle or P/I for 1 h. ChIP were performed as previously described with little modifications [53]. Briefly, chromatin was crosslinked with 1% formaldehyde added to culture medium and incubated at room temperature for 10 min with gentle agitation. Crosslinking was stopped by adding glycine to compose a final 0.125M concentration. Cell were scraped for maximum recovery and washed sequentially with ice-cold PBS-glycine 0.125M and PBS. Nuclei were prepared by sequential incubations on ice for 5 min in 1 ml of buffer A (10 mM Tris-HCl pH 8, 10 mM EDTA, 0.25% Triton X-100, protease inhibitors), and for 30 min in buffer B (10 mM Tris-HCl pH 8, 1 mM EDTA, 200 mM NaCl, protease inhibitors). Nuclei pellets were resuspended in sonication buffer (10 mM Tris-HCl pH 8, 1 mM EDTA, 0.5% SDS, 0.5% Triton X-100, 0.05% NaDOC, 140 mM NaCl) and sonicated to an average 250 bp size using a Branson Digital Sonifier (Branson Ultrasonics). Chromatin was subjected to immunoprecipitation overnight using 20 µl of Protein A and 20 µl of Protein G Dynabeads (Life Technologies) pre-bound with either 3 µg rabbit IgG (Santa Cruz Biotechnology Inc.), 3 µg of H3 (ab1791) or 3 µg of H3K4me1 (ab8895) antibodies from Abcam Inc. Immune complexes were washed sequentially with the following buffers for 2 min at room temperature: Wash B (1% Triton X-100, 0.1% SDS, 150 mM NaCl, 2 mM EDTA, 20 mM Tris-HCl pH 8), Wash C (1% Triton X-100, 0.1% SDS, 500 mM NaCl, 2 mM EDTA, 20 mM Tris-HCl pH 8), Wash D (1% NP-40, 250 mM LiCl, 1 mM EDTA, 10 mM Tris-HCl pH 8), and TEN buffer (50 mM NaCl, 10 mM Tris-HCl pH 8, 1 mM EDTA). Following decrosslink by overnight incubation at 65°C in elution buffer (1% SDS, 50 mM Tris-HCl pH 8, 10 mM EDTA), RNase A and proteinase K treatments, DNA was recovered on QIAquick PCR purification columns (Qiagen). H3K4 monomethylation ChIP enrichment level was measured relative to H3 level by QPCR with Perfecta SYBR green PCR kit (Quanta Bioscience) for known CNS regions, *Ifnγ* promoter and an unrelated negative control (*Pomc* gene promoter) using the following primers: POMC For 5′aggcagatggacgcacataggtaa3′ and Rev 5′tccacttagaactggacagaggct3′. CNS-54kb For 5′ agcctgactggcatattggcaaac 3′ and Rev 5′aaacctgaaggtcgtggcttgact 3′; CNS-34kb For 5′ acttctgaagacaggccacaggtt 3′ and Rev 5′acagctgagactgtggttgacact 3′; CNS-22kb For 5′ ggagatgggaagtcagatcaaag 3′ and Rev 5′ cagaaatttggcctcttaggttt 3′; CNS-6kb For 5′ tgtggacttccattctccacgtca 3′ and Rev 5′ cacgttggttgaactcctggaact 3′; IFNγ prom For 5′ atcacctccattgaagggcttcct 3′ and Rev 5′ ttctcatccacagagcacagcaca.3′. Thereafter H3K4me1/H3 levels were compared between P/I and mock treated cells.

### In vivo killing of MHC class I–deficient cells and m157-Transgenic splenocytes

Splenocytes from B6.H2-/- and m157- transgenic mice were labelled with 0.4 mM CFSE (CFSE low) in RPMI medium containing 5% FCS, and splenocytes from recipient mice were labelled with 4 mM CFSE (CFSE high) in RPMI containing 10% FCS. The splenocytes were then incubated at 37°C for 10 min and then washed three times in RPMI containing 10% FCS. Cells (5×10^6^) of each type were mixed, and the mixture (200 µl) was injected intravenously into recipient mice. After 18 hours, spleens were harvested and red blood cells were lysed. The relative percentage of cells in each CFSE population was measured by FACS as previously described [54].

### Statistical analysis

GraphPad Prism software was used to conduct Student's *t*-test, ANOVA Significance was set at a *P* value of less than 0.05.

## Supporting Information

Figure S1 Expression frequencies of Ly49 receptors in *Ly49h* transgenic mice. Splenocytes of indicated mouse strains were collected and the staining of Ly49A, Ly49C/I/H and Ly49G2 was gaited on CD3^−^DX5^+^ cells. (A) Histograms; (B) quantification 3 mice per group were analyzed. Data are presented as mean ± SEM.
(EPS)

Figure S2 At low dose of MCMV inoculums, A-Ly49h displays a high viral load in target organs. Mice were infected IP with 2500 PFU and the kinetic of viral load in target from indicated strain was determined by PA. 3 to 5 mice per group were analyzed. Data represents one experiment out of 2. Shapes represent statistically significant differences as follows using anova with bonferroni post-test: €: p<0.05 between A-*Ly49h vs* FVB-*Ly49h* or p<0.001 between A- *Ly49h vs* B6. £: p<0.01 between A- *Ly49h vs* FVB-*Ly49h* or p = 0.005 between A- *Ly49h vs* B6 #: p<0.05 between A-*Ly49h vs* B6 and FVB-*Ly49h*, $: p<0.05 between A-*Ly49h vs* B6 or p<0.004 between A- *Ly49h vs* FVB- *Ly49h*, &: p<0.001 between A-*Ly49h vs* FVB-*Ly49h* and B6, *: p<0.05 between A-*Ly49h* vs FVB-*Ly49h vs* and B6.
(EPS)

Figure S3 Proliferation, granzyme and perforin production by NK cells of FVB-*Ly49h* mice in response to low MCMV inoculums. Mice were infected or not with 2500 PFU of MCMV and were sacrificed at indicated days; (A) BrdU incorporation on CD3^−^DX5^+^ Ly49H^+^ NK cells was determined by flow cytometry. (B) MCMV viral titer was quantified by PA in the spleen. (C) Intracellular Granzyme and Perforin expression were analyzed by flow cytometry on CD3^−^DX5^+^ Ly49H^+^ NK cells.
(EPS)

Figure S4 IFNγ production in T cells from RCS mice. (A) Splenocytes were collected from indicated RCS and parental strains and stimulated for 4 h with P/I and the percentage of intracellular IFNγ gaited on CD3^+^DX5^−^ T cells is shown. 3 pooled experiments are shown. (B) Genome-wide linkage analysis was done in the 33 RCS strains shown in Figure 4A using IFNγ production by T cells upon P/I treatment. The negative log genome-wide *P* values are shown.
(EPS)

Figure S5 Similar phenotype and in vivo killing of NK cells from B6 and BcA9 mice. (A) NK cells from B6 and BcA9 were analysed by flow cytometry using indicated cell markers (three mice per group are shown). (B) B6 and BcA9 mice were injected with CFSE labelled splenocytes from MHC-class I deficient and m157-transgenic donors and percent of killing was shown as Ratio MHC-I -/- and m157-Tg versus host (three mice per group are shown).
(EPS)

Figure S6 IFNγ production in NK cells from inbreed strains. (A) Splenocytes or (B) IL-2 derived NK cells from indicated strains were stimulated for 4 h with P/I and the percentage of intracellular IFNγ gaited on CD3^+^DX5^−^ T cells is shown.
(EPS)

Figure S7 NK cell receptor expression in B6, Css10 and BcA9 mice. Indicated NK cell receptors were analysed by FACS from total splenocytes in not infected and infected mice.
(EPS)

Figure S8 IFNγ locus chromatin landscape exhibit multiple novel putative regulatory regions. Genomic regulatory regions are flagged by specific histone post-translational modifications, such as H3K4me1 and H3K4me2. To identify putative IFNγ enhancers, we took advantage of recently published H3K4me2 chromatin immunoprecipitation high-throughput sequences (ChIP-seq) performed in various mouse T cell subsets producing IFNγ and/or IL17 [45]. We retrieved sequence reads mapping under the 6.6 Mbp interval identified by linkage analysis (Figure 5) and identify chromatin region marked with H3K4me2 histone modification using MACS 1.4.1 peak calling algorithm [55]. To generate the sequence read density profile (blue graphs) and to perform peak calling analysis, we used the following parameters: –wig –single-profile –bw 250 –mfold 6,30 –pvalue 1e-5 -g 6600000. Data are shown for 300 kbp surrounding the *IFNγ* gene. The H3K4me2 positive regions identified were summed between the four cell type to obtain a list of putative IFNγ regulatory regions (red bars). These H3K4me2+ regions overlap all known conserved non-coding sequences (CNS; blue bars) and identify novel putative regulatory regions. Mammalian sequence conservation is also shown.
(TIF)

Table S1 NKC and H2 inheritance in the RCS strains.
(PDF)

Table S2 List of genes in the vicinity of chromosome 10 QTL.
(PDF)

Table S3 Exome sequencing analysis in A and BcA9 mice in the vicinity of chromosome 10 QTL.
(PDF)
