# Supplementary figures and images for: Specific Dysregulation of IFNγ Production by Natural Killer Cells Confers Susceptibility to Viral Infection
Source: PLoS Pathog. 2014 Dec 4;10(12):e1004511. doi: 10.1371/journal.ppat.1004511 (PMC4256466; doi:10.1371/journal.ppat.1004511)

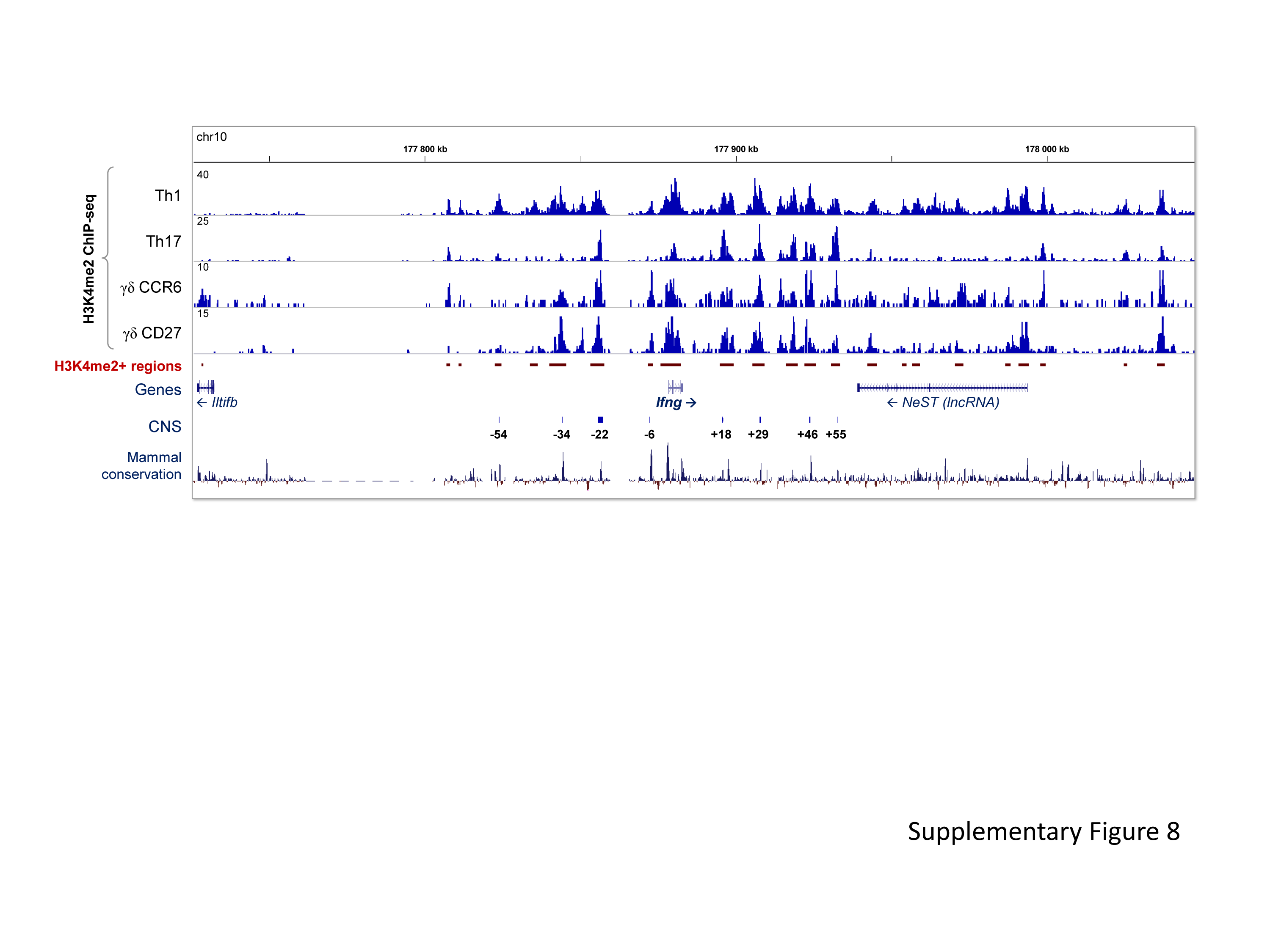

Supplement: Figure S8 — IFNγ locus chromatin landscape exhibit multiple novel putative regulatory regions. Genomic regulatory regions are flagged by specific histone post-translational modifications, such as H3K4me1 and H3K4me2. To identify putative IFNγ enhancers, we took advantage of recently published H3K4me2 chromatin immunoprecipitation high-throughput sequences (ChIP-seq) performed in various mouse T cell subsets producing IFNγ and/or IL17 [45]. We retrieved sequence reads mapping under the 6.6 Mbp interval identified by linkage analysis (Figure 5) and identify chromatin region marked with H3K4me2 histone modification using MACS 1.4.1 peak calling algorithm [55]. To generate the sequence read density profile (blue graphs) and to perform peak calling analysis, we used the following parameters: –wig –single-profile –bw 250 –mfold 6,30 –pvalue 1e-5 -g 6600000. Data are shown for 300 kbp surrounding the IFNγ gene. The H3K4me2 positive regions identified were summed between the four cell type to obtain a list of putative IFNγ regulatory regions (red bars). These H3K4me2+ regions overlap all known conserved non-coding sequences (CNS; blue bars) and identify novel putative regulatory regions. Mammalian sequence conservation is also shown. (TIF) [file ppat.1004511.s008.tif]
