# Supplementary material for: Specific Dysregulation of IFNγ Production by Natural Killer Cells Confers Susceptibility to Viral Infection
Source: PLoS Pathog. 2014 Dec 4;10(12):e1004511. doi: 10.1371/journal.ppat.1004511 (PMC4256466; doi:10.1371/journal.ppat.1004511)
Supplement: Table S1 — NKC and H2 inheritance in the RCS strains. (PDF) [file ppat.1004511.s009.pdf]

Supplementary Table 1: NKC and H2 inheritance in the RCS strains

**AcB Strains**	***NKC***	***H2***
AcB1	A/J	A/J
AcB4	A/J	A/J
AcB5	A/J	A/J
AcB7	A/J	A/J
AcB10	A/J	A/J
AcB11	A/J	A/J
AcB14	A/J	A/J
AcB19	A/J	A/J
AcB20	A/J	A/J
AcB21	A/J	A/J
AcB22	A/J	A/J
AcB23	A/J	B6
AcB29	A/J	A/J
AcB30	A/J	A/J

**BcA Strains**	***NKC***	***H2***
BcA1	B6	B6
BcA2	B6	B6
BcA3	B6	B6
BcA4	B6	A/J
BcA6	B6	B6
BcA7	B6	B6
BcA9	B6	B6
BcA12	B6	B6
BcA14	B6	B6
BcA17	A/J	A/J
BcA18	B6	B6
BcA19	A/J	A/J
BcA20	B6	B6
BcA21	B6	B6
BcA23	B6	B6
BcA24	B6	B6
BcA26	B6	B6
BcA27	A/J	B6
BcA28	B6	B6
BcA29	B6	B6
BcA30	B6	B6
