# Supplementary material for: Specific Dysregulation of IFNγ Production by Natural Killer Cells Confers Susceptibility to Viral Infection
Source: PLoS Pathog. 2014 Dec 4;10(12):e1004511. doi: 10.1371/journal.ppat.1004511 (PMC4256466; doi:10.1371/journal.ppat.1004511)
Supplement: Table S2 — List of genes in the vicinity of chromosome 10 QTL. (PDF) [file ppat.1004511.s010.pdf]

Supplementary Table 2: List of genes in the vicinity of Chromosome 10 QTL

Gene Symbol	Gene name	Start	End	Ref Seq
Trhde	TRH-degrading enzyme	113836078	114238420	NM_146241
Tbc1d15	TBC1 domain family, member 15	114635513	114688521	NM_025706
Rab21	RAB21, member RAS oncogene family	114726917	114752647	NM_024454
Tmem19	transmembrane protein 19	114777795	114799287	NM_133683
Thap2	THAP domain containing, apoptosis associated	114807021	114821491	NM_025780
Tbc1d15	TBC1 domain family, member 15	114635513	114688521	NM_025706
Ccdc131	proline/serine-rich coiled-coil 2	114822014	114869827	NM_001033261
Lgr5	leucine rich repeat containing G protein coupled	114887369	115024836	NM_010195
Tspan8	tetraspanin 8	115254326	115286672	NM_146010
Ptprr	protein tyrosine phosphatase, receptor type, R	115455454	115711985	NM_011217
Kcnmb4	calcium activated potassium channel beta 4	115854923	115910579	NM_021452
Cnot2	CCR4-NOT transcription complex, subunit 2	115922221	115986231	NM_028082
Gm239	hypothetical protein LOC237558	116213600	116333935	NM_001033333
Rab3ip	RAB3A interacting protein	116342839	116387436	NM_001003950
Best3	bestrophin 3	116423369	116462096	NM_001007583
Lrrc10	leucine rich repeat containing 10	116482396	116483824	NM_146242
Cct2	chaperonin subunit 2 (beta)	116488053	116500870	NM_007636
Frs2	fibroblast growth factor receptor substrate 2	116507182	116585530	NM_177798
Yeats4	YEATS domain containing 4	116652197	116654414	NM_026570
9530003J23Rik	hypothetical protein LOC77397	116669292	116675662	NM_029906
Lyzs	lysozyme	116714596	116719328	NM_017372
Cpsf6	cleavage and polyadenylation specific factor 6	116785262	116814005	NM_001013391
Mdm2	transformed mouse 3T3 cell double minute 2	117125960	117147772	NM_010786
Slc35e3	solute carrier family 35, member E3	117170733	117183414	NM_029875
Nup107	nucleoporin 107	117187698	117229761	NM_134010
Rap1b	RAS related protein 1b	117251652	117283030	NM_024457
Mdm1	transformed mouse 3T3 cell double minute 1	117578887	117585195	NM_010785
Il22	interleukin 22	117641997	117647102	NM_016971
Iltifb	interleukin 10-related T cell-derived inducible	117726685	117732094	NM_054079
Ifng	interferon gamma	117878102	117882948	NM_008337
Dyrk2	dual-specificity tyrosine-(Y)-phosphorylation	118296404	118305959	NM_001014390
Grip1	glutamate receptor interacting protein 1 isoform	118891369	119512649	NM_130891
Helb	helicase (DNA) B	119520663	119549946	NM_080446
Irak3	interleukin-1 receptor-associated kinase 3	119578709	119638593	NM_028679
Tmbim4	transmembrane BAX inhibitor motif containing 4	119645881	119661953	NM_026617
1190005P17Rik	hypothetical protein LOC66225	119664115	119669126	NM_025431
Hmga2	high mobility group AT-hook 2	119798330	119913991	NM_010441
Msrb3	methionine-R-sulfoxide reductase B3	120218156	120335908	NM_177092
Lemd3	LEM domain containing 3	120360466	120416386	NM_001081193
Trhde	TRH-degrading enzyme	113836078	114238420	NM_146241
