# Supplementary material for: Specific Dysregulation of IFNγ Production by Natural Killer Cells Confers Susceptibility to Viral Infection
Source: PLoS Pathog. 2014 Dec 4;10(12):e1004511. doi: 10.1371/journal.ppat.1004511 (PMC4256466; doi:10.1371/journal.ppat.1004511)
Supplement: Table S3 — Exome sequencing analysis in A and BcA9 mice in the vicinity of chromosome 10 QTL. (PDF) [file ppat.1004511.s011.pdf]

Supplementary Table 3: Exome Sequencing analysis in A and BcA9 mice in the vicinity of Chromosome 10 QTL

Gene name	Position	Effect	Effect type	Codon change	Amino acid change	Gene type	B6 reference allele	Altred alleles	db_snp_id	Presence in other strains	T cells	NK cells
Trhde	113838413	UTR_3_PRIME				protein _coding	A	T	rs29341542		+	+
Tph2	114516680	UTR_3_PRIME				protein _coding	ACCCCCC	ACCCCC CC	.		+	+
Tph2	114516850	NON_SYNONYMO US_CODING	MISSENSE	cCc/cGc	P447R	protein _coding	G	C	rs33849125	DBA/2		
Tbc1d15	114636486	UTR_3_PRIME				protein _coding	ATCAGTACA CTGTCTC	ATC	.		+/-	+/-
Tbc1d15	114636536	UTR_3_PRIME				protein _coding	C	T	rs46784319	DBA/2, 129/S1		
Tbc1d15	114636539	UTR_3_PRIME				protein _coding	G	A	rs48390084	DBA/2, 129/S1		
Tbc1d15	114639607	NON_SYNONYMO US_CODING	MISSENSE	Ata/Gta	I586V	protein _coding	T	C	rs29344612	DBA/2, 129/S1		
Tspan8	115254596	UTR_5_PRIME				protein _coding	A	C	rs29349889	DBA/2		
Tspan8	115286550	UTR_3_PRIME				protein _coding	G	T	rs13473211	DBA/2		
Cpsf6	116814084	UPSTREAM				protein _coding	G	T	.		+/-	+/-
Cpsf6	116817914	UPSTREAM				protein _coding	AGGTGGTG GTGGTGGT GGTGGTGG TGGTGGTG GTGGTG	AGGTG GTGGTG GTGGTG GTGGTG GTGGTG	.		+/-	+/-
Cpm	117066546	UPSTREAM				protein _coding	A	G	rs33880972	DBA/2		
Mdm1	117595588	NON_SYNONYMO US_CODING	MISSENSE	gAc/gGc	D437G	protein _coding	A	G	rs52684110	DBA/2, 129/S1, C3H		
Mdm1	117601369	CODON_CHANGE _PLUS_CODON_I NSERTION		gat/gaGGG t	D572EG	protein _coding	A	AGGG	.		+/-	+/-
Il22	117641848	UPSTREAM				protein _coding	G	A	rs49141619	DBA/2		
Il22	117641875	UPSTREAM				protein _coding	C	A	.		++	+/-
Il22	117641885	UPSTREAM				protein _coding	G	A	rs46351844	DBA/2		
Il22	117641918	UPSTREAM				protein _coding	T	G	rs50679834	DBA/2		
Il22	117641956	UPSTREAM				protein _coding	C	T	.		++	+/-
Il22	117641959	UPSTREAM				protein _coding	A	G	.		++	+/-
Il22	117642043	UTR_5_PRIME				protein _coding	T	G	.	DBA/2		
Il22	117642044	START_GAINED				protein _coding	C	G	rs48127970	DBA/2, 129/S1, C3H		
Il22	117642690	NON_SYNONYMO US_CODING	MISSENSE	aGt/aAt	S84N	protein _coding	G	A	rs29355558	DBA/2		
Il22	117646696	UTR_3_PRIME				protein _coding	T	C	rs29329802	DBA/2, 129/S1		
Iltifb	117731402	NON_SYNONYMO US_CODING	MISSENSE	aGt/aAt	S84N	protein _coding	C	T	rs50848176	DBA/2		
Iltifb	117731940	NON_SYNONYMO US_CODING	MISSENSE	Atc/Gtc	I36V	protein _coding	T	C	rs46916394	129/S1, C3H		
Iltifb	117732067	UTR_5_PRIME				protein _coding	A	C	.		+	+
Iltifb	117732097	UPSTREAM				protein _coding	C	T	rs50966058	DBA/2		
4932442 E05Rik, Dyrk2	118298244	FRAME_SHIFT				protein _coding	AGGGGGGG G	AGGGG GGG	.		+	+
Grip1	119334821	NON_SYNONYMO US_CODING	MISSENSE	Gca/Aca	A36T	protein _coding	G	A	rs52029579	DBA/2		
